# St. Luke’s Hospital

**Published:** 1869-10

**Authors:** Jno. E. Owens

**Affiliations:** 112 Randolph Street


ARTICLE II.—*St. Luke's Hospital*, under the care of JNO. E. OWENS, M.D.

*A Case of Pertussis.*—May J.; aged 6 years; admitted June 30, 1869, five weeks previous to which, according to the mother's account, she was attacked with pertussis. How long she had had the characteristic cough is unknown; but, upon admission, it was pathognomonic. The paroxysms, which were more frequent at night, occasioned a regurgitation of almost every thing that was eaten. Before instituting any treatment in this disease, one must ascertain not only the degree of violence of the paroxysms, but also their number during twenty-four hours, or during any stated period of sufficient length. In order to determine the latter, we have adopted the suggestion of Trousseau, of sticking a pin-hole in a card for every paroxysm. Since, during the night, more or less of the paroxysms would be lost, the nurse was instructed to count from 6 A.M. to 10 P.M. The accompanying table — an exact copy of the dated card — gives the number of paroxysms during this period of the day, except the first record, the admission day, which was counted from 11 A.M. to 10 P.M.:

June 30, 1869, 28 paroxysms; July 1, 1869, 26; July 2, 1869, 15; July 3, 1869, 18; July 4, 1869, 15; July 5, 1869, 15; July 6, 1869, 13; July 7, 1869, 10; July 8, 1869, 10; July 9, 1869, 11; July 10, 1869, 8; July 11, 1869, 9; July 12, 1869, 6; July 13, 1869, 8; July 14, 1869, 3.

The patient was put upon the following treatment, July 2nd: Atropia sulph. gr. $\frac{1}{150}$, aqua f ʒ ij. S. To be given each morning, fasting. This dose was continued till July 7th, when an additional drachm was administered.

July 6th, about an ounce, only, of dark, offensive urine passed. From this date to the 8th, at two P.M., there was almost an entire suppression, except the occasional passage of a drachm, probably, during a paroxysm.

The patient took no atropia after the 7th. Sweet spirits nitre was given every two hours during the day (7th). Its exhibition was followed by a copious diaphoresis, but the medicine had no effect upon the kidneys. The perspiration imparted to the clothing a strong, urinous smell. The nitre, however, produced vomiting towards evening, when elaterium, gr. $\frac{1}{20}$, (Clutterbuck,) was ordered every two hours. Six doses were administered without effect, when the dose was doubled, which was followed by watery discharges, with a pretty free secretion of urine. During the suppression there was an absolute freedom from pain; neither was there drowsiness, or dryness of the throat. Ever since the exhibition of atropia, the pupils have been moderately dilated, and when it was discontinued the dilatation passed away, to some extent, but temporarily, for two or three times since the 7th the dilatation became excessive, and vision, in a degree, interfered with.

July 19. — The cough, occasionally, has spasmodic quality, but it recurs so seldom that the case seems to require no further treatment. She goes out to-day, feeling very well.

Hooping-cough, although a disease requiring very careful treatment, in many cases, is an affection for which almost every old woman has her specific. In speaking of what seems to be a very successful mode of treatment, I can not do better than quote the words of the lamented Trousseau: "The active principle of solonaceous plants influences neuroses only when given in sufficiently large doses, and this influence lasts for some time; but lest the therapeutic effects should be greater than desired, the medicine should first be given in doses which are probably less than those needed for exerting a favorable action on the disease; these doses must be gradually increased until therapeutic effects begin to show themselves. As soon as this result is obtained, it is generally sufficient to continue the same daily dose, in order to increase the good effect produced. If the dose which has brought on these good results, were increased hastily, with the view of accelerating the cure, and especially if it were repeated on the same day, one might, at first, wonder at the success obtained; but unpleasant dryness of the fauces, and some disturbance of vision, which increases rapidly, would soon render a diminution of the dose necessary, and the consequence of this would be to allow the disease to reproduce itself, and to escape the influence of the mode of treatment."

The case involves several points of interest, viz.:

The marked influence which the drug seemed to possess, of lessening both the number and violence of the paroxysms; the suppression of urine — probably the effect of atropia; the efficiency of elaterium in re-establishing the renal secretion, in addition to its action upon the bowels; the copious perspiration, which imparted a urinous smell to the clothing, and which depurated the blood to a great extent, thereby protecting the nervous system; lastly, the excessive dilatation of the pupils, which occurred a number of times from the 7th to the 11th, seems to prove that the influence exerted by the salt, and possibly by solanaceous plants in general, lasts for some time, and we are justified in concluding that inasmuch as its physiological manifestation could be seen during this time, it was likewise making itself felt upon the neurosis.

112 *Randolph Street.*

